# Metabolomics Analysis Revealed That SXZ Ameliorates Cognitive Impairment in Naturally Aged Rats by Promoting Glutathione Metabolism and Inhibiting the Ferroptosis Pathway

**DOI:** 10.1002/cns.71194

**Published:** 2026-10-07

**Authors:** Mengya Liu, Shunyi Li, Yonglin Liang, Yugui Zhang, Xutong Jia, Yongqiang Duan, Min Bai

**Affiliations:** ^1^ Ningxia Medical University Yinchuan Ningxia China; ^2^ Gansu University of Chinese Medicine Lanzhou Gansu China

**Keywords:** age‐related cognitive impairment, ferroptosis, glutathione metabolism, metabolomics, Shenxian Zhou

## Abstract

**Objective:**

This study aimed to examine the potential neuroprotective effects of SXZ on cognitive impairment in naturally aging rats and to explore the underlying molecular mechanisms.

**Methods:**

Eighteen‐month‐old Sprague–Dawley rats were used as a natural aging model and administered Shenxian Zhou (SXZ) for 6 months. Cognitive function was assessed using behavioral evaluations. Hematoxylin–eosin, Nissl, and immunofluorescence staining for NeuN were performed to evaluate neuronal pathology in aging rats. Metabolomics analysis was performed to characterize hippocampal metabolites, followed by molecular validation.

**Results:**

SXZ attenuated age‐related cognitive decline and reduced hippocampal pathological and neuronal damage in naturally aging rats. SXZ‐H significantly modulated glutathione metabolism and ferroptosis‐associated signaling pathways. Transmission electron microscopy revealed mitochondrial morphological changes consistent with ferroptosis in hippocampal neurons of naturally aging rats. Molecular biology experiments further indicated that, subsequent to SXZ‐H intervention, lipid peroxidation and oxidative stress levels were significantly reduced. Furthermore, expression levels of p53 and Keap1 genes and proteins were significantly downregulated, whereas the expression of SLC7A11, GPX4, Nrf2, and HO‐1 genes and proteins was notably upregulated.

**Conclusion:**

SXZ‐H effectively mitigates cognitive impairment in naturally aging rats, an effect that may be mediated by promoting glutathione metabolism and regulating key ferroptosis‐related proteins, thereby inhibiting neuronal ferroptosis.

## Graphical abstract

In aged rats, 6‐month SXZ treatment reversed spatial memory deficits and preserved hippocampal integrity. Mechanistic assays show that SXZ promotes glutathione metabolism and inhibits ferroptosis via p53/SLC7A11/GPX4 and Keap1/Nrf2/HO‐1 pathways. SXZ is a novel neuroprotective agent against age‐related cognitive decline.

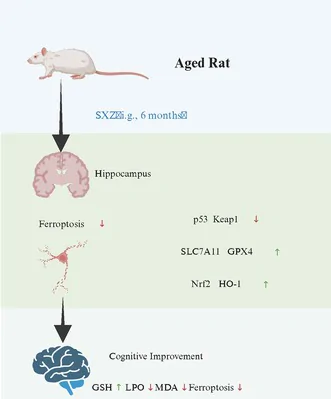

## 1 Introduction

Aging is a natural biological process characterized by the progressive decline of tissues and organs [1]. As the global population ages, the risk of developing age‐related diseases increases significantly [2]. Aging impairs learning and memory functions and contributes to cognitive decline [3]. Epidemiological studies estimate that 10%–20% of individuals aged ≥ 65 years experience cognitive decline [4], substantially increasing the risk of dementia and other neurological diseases. Age‐related cognitive impairment also imposes considerable burdens on families and society. Currently available pharmacological treatments for age‐related cognitive impairment remain limited and often unsatisfactory. Systematic reviews have reported that acetylcholinesterase inhibitors and memantine provide limited benefit in preventing dementia among patients with mild cognitive impairment and may cause adverse effects [5, 6]. Accordingly, safer and more effective therapeutic strategies are needed.

Traditional Chinese medicine (TCM) has a long history of clinical use in the management of age‐related cognitive impairment. Systematic reviews and meta‐analyses suggested that TCM may delay cognitive deterioration and enhance cognitive function in patients with Alzheimer's disease (AD) [7]. Shenxian Zhou (SXZ), initially documented in the Dunhuang Manuscript P.3810 Hu Xi Jing Gong Miao Jue (“Essential Methods of Breathing and Meditation” in Chinese), comprises four herbal ingredients: *Oryza sativa* L., Dioscoreae Rhizoma, Allii Tuberosi Semen, and Euryales Semen. Tohda C. et al. [8], reported that diosgenin, a principal component of Dioscoreae Rhizoma, improved cognitive function in healthy adults. Chiu C.S. et al. [9], reported that Dioscoreae Rhizoma ameliorated cognitive deficits in aged mice by enhancing endogenous antioxidant enzyme activity. Ye J.Y. et al. [10], discovered that β‐sitosterol, an active constituent of Allii Tuberosi Semen, enhanced cognitive function by improving learning and memory impairments in APP/PS1 mice, correlating with decreased Aβ deposition. Vidal V. et al. [11], revealed that oleic acid, a major active compound of Euryales Semen, facilitated synaptic maturation and improved cognitive performance in the Ts65Dn mouse model of Down syndrome. Earlier studies confirmed that SXZ exhibits anti‐aging effects [12] and exerts significant neuroprotective activity against X‐ray‐induced brain injury in rats [13]. However, the therapeutic efficacy and underlying mechanisms of SXZ in treating age‐related cognitive impairment remain unclear.

Metabolomics, a branch of omics research, is the comprehensive study of the end products of metabolic processes and provides unique advantages in enhancing the understanding of physiological and pathological phenomena [14]. Untargeted metabolomics systematically analyzes the dynamic changes of small‐molecule metabolites in organisms, thereby revealing global imbalances in metabolic networks during pathological processes [15]. It has demonstrated unique advantages in clarifying the mechanisms underlying age‐related cognitive impairment. For instance, He J. et al. [16], identified that L‐arginine metabolic dysregulation was closely associated with age‐related cognitive impairment through plasma untargeted metabolomics analysis. Dong Y. et al. [17], utilized untargeted ultra‐performance liquid chromatography–tandem mass spectrometry to investigate age‐dependent metabolic alterations in the hippocampus of non‐transgenic (NTg) and triple‐transgenic (3xTg‐AD) mice, and reported that pathways related to energy production, fatty acid oxidation, glutamate metabolism, and sphingolipid metabolism were significantly involved in NTg and 3xTg‐AD brains. No study has examined the molecular mechanism by which SXZ alleviates aging‐related cognitive decline using metabolomics; therefore, this study aimed to investigate the protective effects of SXZ against cognitive impairment in naturally aging rats and, by employing metabolomics, to elucidate the potential molecular mechanisms by which SXZ mitigates age‐related cognitive decline.

## 2 Materials and Methods

### 2.1 Animals

Female Sprague–Dawley rats, aged 2 months and 18 months and weighing approximately 220 g, were procured from Gansu University of Chinese Medicine (Gansu, China) and housed under specific pathogen‐free conditions. The rats were maintained at 22°C with 45%–55% relative humidity under a 12‐h light/dark cycle, with free access to food and water.

### 2.2 SXZ Intervention Protocol

Following model validation, 12 two‐month‐old rats were assigned to the control group (Control), and 48 eighteen‐month‐old rats were randomly allocated into four groups (12 rats per group): Model control group (Model), SXZ high‐dose group (SXZ‐H, 20 g/kg), SXZ medium‐dose group (SXZ‐M, 10 g/kg), and SXZ low‐dose group (SXZ‐L, 5 g/kg) [18]. Control and Model group rats received equal volumes of normal saline by oral gavage, whereas those in the treatment groups received SXZ at specified doses. Treatment continued for 6 months. SXZ preparation is described in Supporting Information S1.

### 2.3 Analysis of Bioactive Components of SXZ in Serum

High‐performance liquid chromatography–tandem mass spectrometry (HPLC‐MS/MS) technology was used to identify the bioactive constituents of SXZ that entered the bloodstream and to assess preparation quality and stability. Shanghai Meiji Biomedical Technology Co. Ltd. facilitated the execution of the HPLC‐MS/MS analysis. Supporting Information S1 presents the preparation of drug‐infused serum and specific parameters for HPLC‐MS/MS analysis.

### 2.4 Behavioral Tests

Y maze [19] and Morris water maze [20] were used to assess the spatial learning and memory capabilities of rats. Detailed procedures are provided in Supporting Information S1.

### 2.5 Sample Collection and Processing

After behavioral testing, all rats were deeply anesthetized and euthanized. For each experimental group, three rats were randomly selected using a random number table; their whole brains were harvested and fixed in 4% paraformaldehyde for paraffin embedding and subsequent histopathological and immunofluorescence analyses. Brains from another three rats per group were rapidly frozen in liquid nitrogen for ROS analysis. Hippocampi from the remaining six rats per group were dissected on ice. A 1 mm^3^ tissue block from each hippocampus was fixed in electron microscopy fixative, whereas the residual hippocampal tissue was rinsed with phosphate‐buffered saline (PBS), snap‐frozen in liquid nitrogen, and stored at −80°C for further analyses. All procedures were performed on ice and completed as quickly as possible to minimize tissue degradation.

### 2.6 Histopathological Observation of the Hippocampus

#### 2.6.1 Hematoxylin–Eosin (HE) Staining

Brain tissues fixed in 4% paraformaldehyde were routinely paraffin‐embedded and sectioned, followed by HE staining. Hippocampal pathological changes were examined using a light microscope.

#### 2.6.2 Nissl Staining for Neuronal Morphology

Paraffin‐embedded sections were subjected to Nissl staining after fixation in 4% paraformaldehyde. Following dehydration and mounting, the sections were examined and photographed using a light microscope. For each animal, three coronal sections were examined, and three randomly selected high‐power fields (20×) from each section were evaluated. Nissl bodies were counted and averaged. To ensure double‐blind conditions, all sections were coded by a third party, and both the technician performing the staining and the investigator performing the quantification were blinded to group allocation until all data had been compiled and analyzed. The quantity of Nissl bodies in hippocampal CA1, CA3, and DG regions was assessed using ImageJ analysis software.

### 2.7 Untargeted Metabolomics Testing and Analysis

Rat hippocampal tissues were rinsed with PBS, rapidly frozen in liquid nitrogen, and stored at −80°C until further untargeted metabolomic analysis. Six hippocampal tissue samples from each of the Control, Model, and SXZ‐H groups were analyzed by untargeted metabolomics at Panomics (Suzhou) Biomedical Technology Co. Ltd. The metabolomics data have been deposited in the MetaboLights [21] repository with the study identifier MTBLS15736. Detailed procedures are provided in Supporting Information S1.

### 2.8 Transmission Electron Microscopy for Mitochondrial Morphology

Hippocampus tissue fixed in electron microscope fixation solution (Servicebio, G1102) was washed with phosphate buffer, post‐fixed with osmium tetroxide, dehydrated through graded ethanol dehydration, embedded, and polymerized after replacement with 100% acetone. Ultrathin sections were prepared, routinely stained, thoroughly dried, and examined under a transmission electron microscope (Hitachi, Japan) to assess mitochondrial morphological changes. Representative images were captured for analysis.

### 2.9 Immunofluorescence Histochemistry for Protein Expression in Hippocampal Neurons

Paraffin‐embedded hippocampal sections were washed in PBS and incubated with diluted primary antibody, including NeuN (Servicebio, GB15138), 4‐HNE (GeneTex, GTX17571), p53 (CST, CST2524), SLC7A11 (GeneTex, GTX04907), GPX4 (GeneTex, GTX54095), Keap1 (GeneTex, GTX54329), Nrf2 (GeneTex, GTX103322), and HO‐1 (GeneTex, GTX101147). In a humidified environment at 37°C, sections were incubated in the dark for 30 min, subsequently washed with PBS, and treated with secondary antibodies at room temperature for 1 h. Following an extra PBS wash, DAPI staining was performed for 10 min in the absence of light. Ultimately, slides were prepared and imaged using a laser confocal microscope. For each section, three non‐overlapping visual fields were captured, and the fluorescence intensity was semiquantitatively analyzed using ImageJ software. The mean value of these three fields was calculated for each section.

### 2.10 Fluorescence Staining for ROS Expression in Hippocampal Tissue

Frozen hippocampal sections were incubated with a diluted solution of the BBoxiProbe DHE reactive oxygen species fluorescent probe (BestBio, BB‐470516) for 30 min. After washing, sections were mounted with an anti‐fade medium containing DAPI (Servicebio, EE0015). Fluorescence signals were visualized using a laser confocal microscope. Three non‐overlapping fields per section were captured and semiquantitatively analyzed using ImageJ software, with the mean value calculated for each section.

### 2.11 Colorimetric Assays for Fe^2+^, SOD, MDA, LPO, GSH, GSSG, GSH‐Px, NADP ^+^, and NADPH Levels in the Hippocampus

Frozen hippocampal tissue was obtained, and commercial assay kits were utilized to evaluate Fe^2+^ concentration (Elabscience, E‐BC‐K773‐M), SOD (Nanjing Jiancheng, A001‐1‐2), MDA (Nanjing Jiancheng, A003‐1‐2), LPO (Nanjing Jiancheng, A106‐1‐3), T‐GSH/GSSG (Nanjing Jiancheng, A061‐1‐2), GSH‐Px (Nanjing Jiancheng, A005‐1‐2), and NADPH/NADP^+^ (LeadBio, LDQB‐E‐1133) in rat hippocampal tissue. All procedures were executed in strict accordance with the manufacturer's instructions.

### 2.12 Quantitative Real‐Time Polymerase Chain Reaction (RT‐qPCR) for mRNA Expression of p53, SLC7A11, GPX4, Keap1, Nrf2, and HO‐1

The Total RNA Kit (Yeasen, 19221ES50) was used for RNA extraction from frozen hippocampal tissue, followed by cDNA synthesis using the Reverse Transcript Premix (Yeasen, H9405020). The Amplification Premix (Yeasen, H6412070) was utilized to amplify p53, SLC7A11, GPX4, Keap1, Nrf2, HO‐1, and the internal control GAPDH. We performed RT‐qPCR and evaluated the relative gene expression levels using the 2^−ΔΔCt^ method. Table S1 presents the relevant primer sequences.

### 2.13 Western Blot (WB) Analysis of Protein Expression in Hippocampal Tissue

Protein was extracted from hippocampal tissue, and concentrations were quantified using the bicinchoninic acid method. Equal quantities of protein were electrophoresed, transferred to membranes, and subsequently blocked, followed by washing with TBST. Membranes were incubated overnight at 4°C with primary antibodies against p53 (CST, CST2524), SLC7A11 (GeneTex, GTX04907), GPX4 (GeneTex, GTX54095), Keap1 (GeneTex, GTX54329), Nrf2 (GeneTex, GTX103322), and HO‐1 (GeneTex, GTX101147), all diluted to 1:500, along with GAPDH (Zen Bioscience, 200,306‐7E4) at a dilution of 1:5000 as the internal control. After washing, membranes were incubated with secondary antibodies for 1 h. Protein signals were generated via enhanced chemiluminescence, imaged automatically, and quantified using ImageJ, with GAPDH as the loading control for normalization.

### 2.14 Statistical Analysis

Statistical analysis of experimental results was conducted using GraphPad Prism software (version 9.5). Before analysis, tests for data normality and homogeneity of variance were performed. The mean ± standard deviation (SD) was calculated if the experimental data conformed to the normal distribution. Comparisons between two groups were conducted using Student's *t*‐test, whereas comparisons among three or more groups were executed using one‐way analysis of variance (ANOVA), followed by Tukey's post hoc test for homogeneous variances or Dunnett's T3 post hoc test for heterogeneous variances. Comparisons between various groups at multiple time intervals were assessed using two‐way ANOVA. For data that deviated from a normal distribution, group comparisons were conducted using the rank‐sum test. A *p* < 0.05 was considered statistically significant.

## 3 Results

### 3.1 Analysis of Bioactive Constituents of SXZ in Serum

The mass spectrometry analysis of SXZ‐medicated serum produced comprehensive ion fragmentation data, which was matched against the proprietary MJBIOTCM (Meiji Biological TCM Metabolite Database) and a local library. We identified 90 absorbed constituents, including lipids, steroids, and steroid derivatives, organic acids and their derivatives, and flavonoids. Figure S1 depicts the distribution of compound classes by content and quantity. Based on relative peak area, the top 10 bioactive constituents were identified as Pc(34:2), Oroxylin A, Wogonin, Galangin 3‐methyl ether, methyl 3‐(3,4‐dihydroxyphenyl) propanoate, Paederoside, Oroxyloside, Verbasoside, Sinensetin, and 2‐methyl‐6‐phenyl‐4H‐pyran‐4‐one. Table S2 presents detailed information.

### 3.2 Protective Effect of SXZ on Age‐Related Cognitive Impairment in Rats

Y‐maze and MWM tests were employed to assess whether SXZ may alleviate cognitive impairments in naturally aging rats. Figure 1A depicts the schematic representation of the Y‐maze. Compared to the Control group, model rats exhibited a significantly diminished spontaneous alternation rate, which was notably restored after 6 months of SXZ‐H and SXZ‐M treatment. (*p* < 0.05, Figure 1B), demonstrating that SXZ enhanced spatial exploration capabilities in naturally aging rats.

**FIGURE 1:**
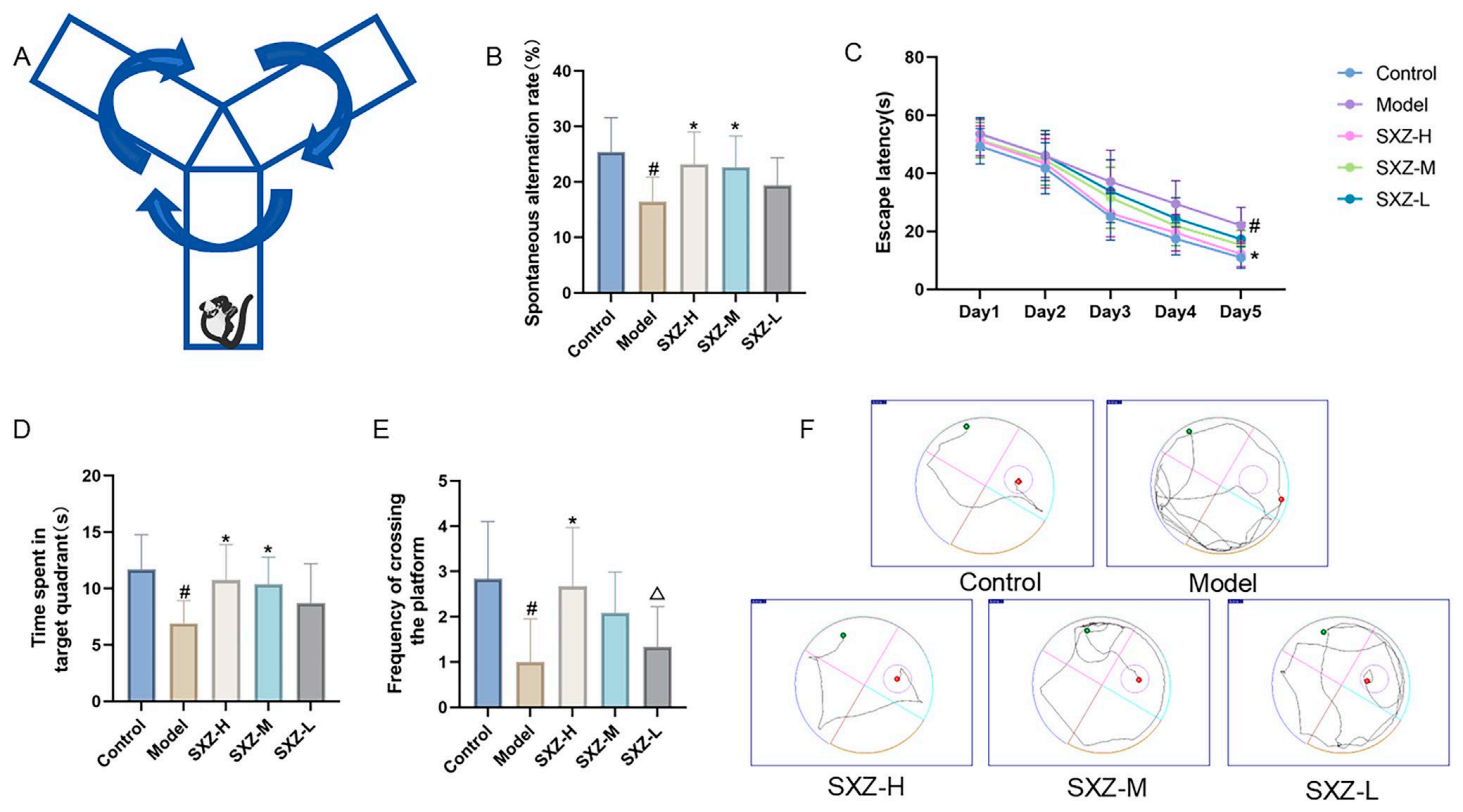
Protective effects of SXZ on cognitive impairment in naturally aging rats. (A) Schematic diagram of the Y‐maze. (B) Effect of SXZ on spontaneous alternation rate in each group (*n* = 12). (C) Effect of SXZ on escape latency across groups (*n* = 12). (D) Effect of SXZ on time spent in the target quadrant (*n* = 12). (E) Effect of SXZ on the number of platform crossings (*n* = 12). (F) Representative swimming paths recorded by the MWM system. Values are expressed as mean ± SD. ^#^ *p* < 0.05 versus Control; *p < 0.05 versus Model. ^△^ *p* < 0.05 versus SXZ‐H.

To evaluate the impact of SXZ on spatial learning and memory, rats underwent the Morris water maze test. In the MWM assessment, escape latency consistently diminished throughout the training days in all groups. On day 5, the Model rats exhibited a significantly prolonged escape latency compared to the Control group. Compared to Model rats, SXZ‐H rats exhibited a significantly reduced duration to identify the concealed platform on day 5 (Figure 1C). On day 6 of the probe trial, Model rats exhibited a marked decrease in platform crossings and spent significantly less time in the target quadrant compared to Controls. Conversely, SXZ‐H rats exhibited a significant increase in both platform crossings and duration spent in the target quadrant compared to the Model group (Figure 1D–F). Additionally, the number of times SXZ‐L crossed the platform decreased significantly compared to SXZ‐H. Collectively, these findings indicate that SXZ enhances spatial cognitive abilities and safeguards naturally aging rats from age‐associated cognitive decline, with SXZ‐H exhibiting the most significant effect.

### 3.3 Effects of SXZ‐H on Hippocampal Histopathology in Naturally Aging Rats

Neuronal impairment contributes significantly to cognitive deterioration. HE staining revealed that cells in the model rats were loosely and irregularly organized, with some exhibiting swelling, cytoplasmic hyperchromasia, and nuclear pyknosis. These pathological changes were attenuated following SXZ‐H treatment (Figure 2A), indicating that after 6 months of intervention, SXZ‐H mitigated hippocampal pathological damage in naturally aging rats. To further investigate the protective effects of SXZ‐H on hippocampal neurons, NeuN immunofluorescence staining, as a neuronal marker, was performed in combination with Nissl staining. The findings revealed a significant decrease in NeuN‐positive neurons in Model rats, whereas SXZ‐H treatment notably mitigated this decline (Figure 2B). Nissl staining revealed that hippocampal neurons in Model rats exhibited a sparse configuration with indistinct boundaries, and the quantity of Nissl^+^ neurons was significantly diminished. These changes were markedly attenuated following SXZ‐H treatment (Figure 2C). The quantification of NeuN‐positive neurons and Nissl bodies among groups is illustrated in Figure 2D–G.

**FIGURE 2:**
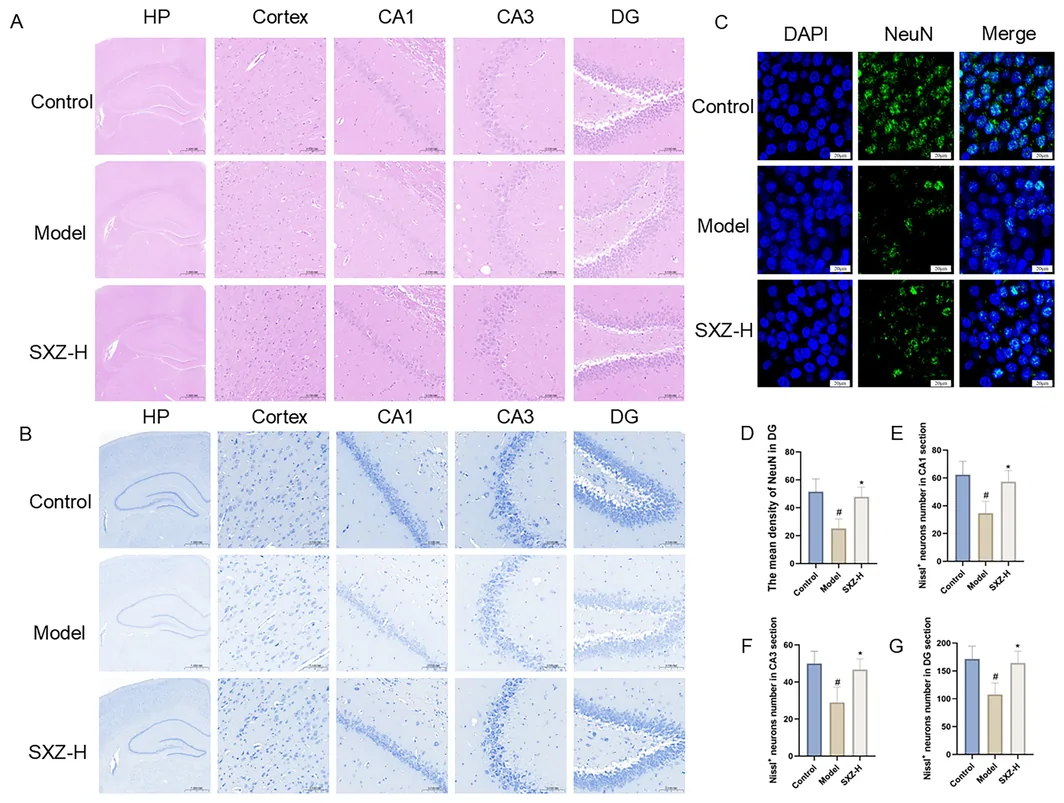
SXZ‐H mitigates hippocampal neuronal damage in naturally aging rats. (A) H/E staining (HP 2×, scale bar = 1000 μm, Cortex, CA1, CA3, DG 20×, scale bar = 100 μm). (B) NeuN expression (immunofluorescence, 100×, scale bar = 20 μm). (C) Nissl staining (HP 2×, scale bar = 1000 μm, Cortex, CA1, CA3, DG 20×, scale bar = 100 μm). (D) Average NeuN density in the DG region (*n* = 3). (E–G) Number of Nissl‐positive neurons in CA1, CA3, and DG regions (*n* = 3). Data are presented as mean ± SEM. ^#^ *p* < 0.05 versus Control; *p < 0.05 versus Model.

### 3.4 Effects of SXZ‐H on Hippocampal Metabolic Profiles in Rats With Age‐Related Cognitive Impairment

The OPLS‐DA score plot (Figure 3A) reveals clear separation between groups, indicating significant intergroup variations and suggesting that natural aging significantly influenced endogenous metabolites in the hippocampus in positive and negative ion modes, whereas 6 months post‐SXZ‐H exhibited a significant modulatory effect. The comprehensive metabolite clustering heatmap demonstrated distinct separation between the Model group and the Control and SXZ‐H groups (Figure 3B). Subsequently, KEGG enrichment and Z‐score assessment of differential metabolites were performed, indicating significant enrichment of these metabolites in multiple pathways associated with lipid peroxidation and redox regulation, including arachidonic acid metabolism, unsaturated fatty acid synthesis, and glutathione metabolism (Figure 3C,D). These pathways are functionally comparable to ferroptosis. Consequently, we focused on glutathione metabolism and ferroptosis pathways for comprehensive analysis and visually examined the relative concentrations of the two differential metabolites. The visualization of the relative abundance of differential metabolites revealed that two metabolites—glutathionylspermine and coenzyme Q10—were upregulated following SXZ‐H treatment in naturally aging rats, with glutathionylspermine exhibiting a significant increase (*p* < 0.05). Conversely, eight metabolites exhibited downregulation, namely Fe^2+^, glutathionylspermidine, mevalonic acid, L‐glutamic acid, α‐tocopherol, iron, NADP^+^, and L‐ornithine, with Fe^2+^, glutathionylspermidine, iron, L‐ornithine, mevalonic acid, and NADP^+^ indicating significant reductions (*p* < 0.05, Figure 3E). The findings mentioned above suggest that SXZ‐H may attain its therapeutic efficacy by modulating the levels of critical differential metabolites.

**FIGURE 3:**
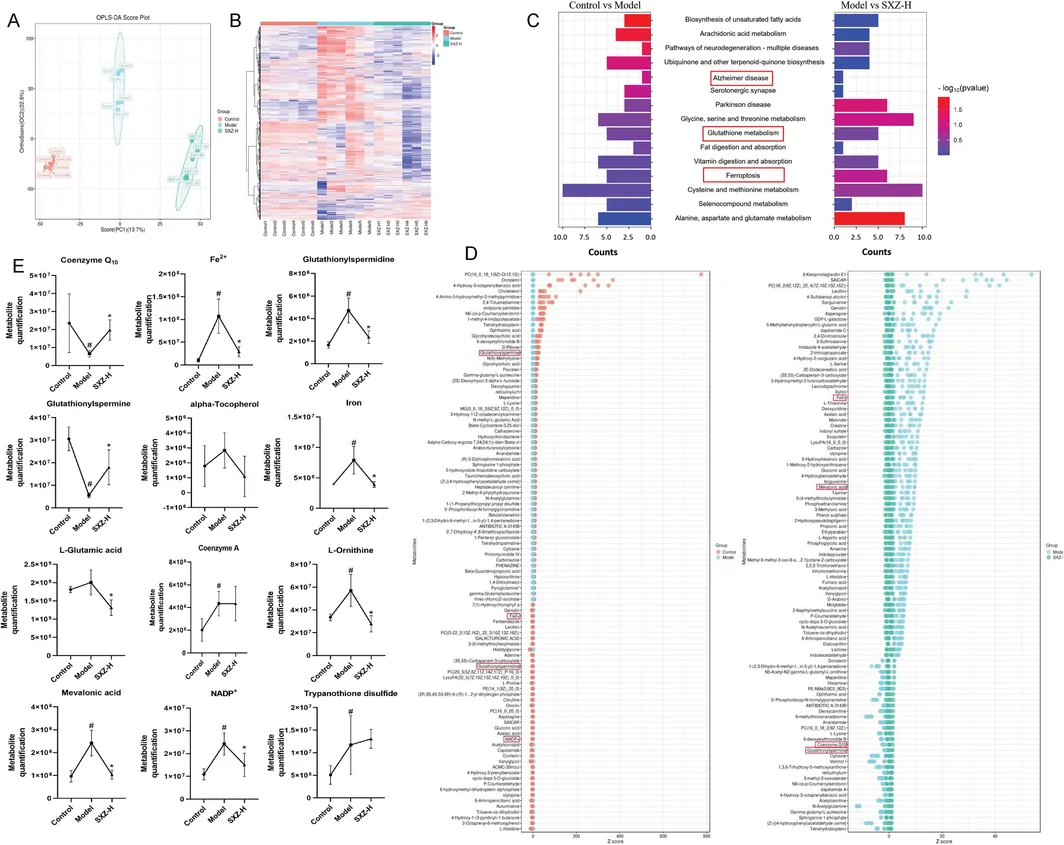
SXZ‐H alters hippocampal metabolomic profiles in aging rats. (A) OPLS‐DA score plots of each group. (B) Hierarchical clustering heatmap of overall hippocampal metabolites. (C) KEGG enrichment pathways. (D) Z‐score plots of differential metabolites between Control versus Model and Model versus SXZ‐H. (E) Relative abundance of differential metabolites in the glutathione metabolism and ferroptosis pathways (*n* = 6). Data are presented as mean ± SD. ^#^ *p* < 0.05 versus Control; *p < 0.05 versus Model.

### 3.5 Effects of SXZ‐H on Ferroptosis‐Related Indicators in the Hippocampus of Naturally Aging Rats

Metabolomics analysis revealed that the protective effect of SXZ‐H against age‐related cognitive decline may be associated with ferroptosis. Consequently, TEM was employed to examine mitochondrial morphology in hippocampal neurons to evaluate the occurrence of ferroptosis in naturally aging rats. Figure 4A demonstrates that hippocampal neurons from Model rats exhibited characteristic ferroptotic features, including mitochondrial shrinkage, elevated double‐membrane density, and reduced cristae. The SXZ‐H treatment significantly mitigated these abnormalities. These results indicate that ferroptosis may transpire in hippocampal neurons of naturally aging rats. Lipid peroxidation and oxidative stress levels were assessed in hippocampal tissue to further validate ferroptosis. The concentrations of 4‐HNE, Fe^2+^, LPO, MDA, ROS, and the NADP^+^/NADPH ratio were significantly increased in the Model group, whereas the activities of GSH‐Px, SOD, and the GSH/GSSG ratio were notably diminished. These indicators indicated significant enhancement, with the exception of Fe^2+^, after 6 months of SXZ‐H treatment (Figure 4B–L). These findings revealed that SXZ‐H mitigates oxidative damage by decreasing lipid peroxidation and augmenting antioxidant defense, inhibiting neuronal ferroptosis in the hippocampus.

**FIGURE 4:**
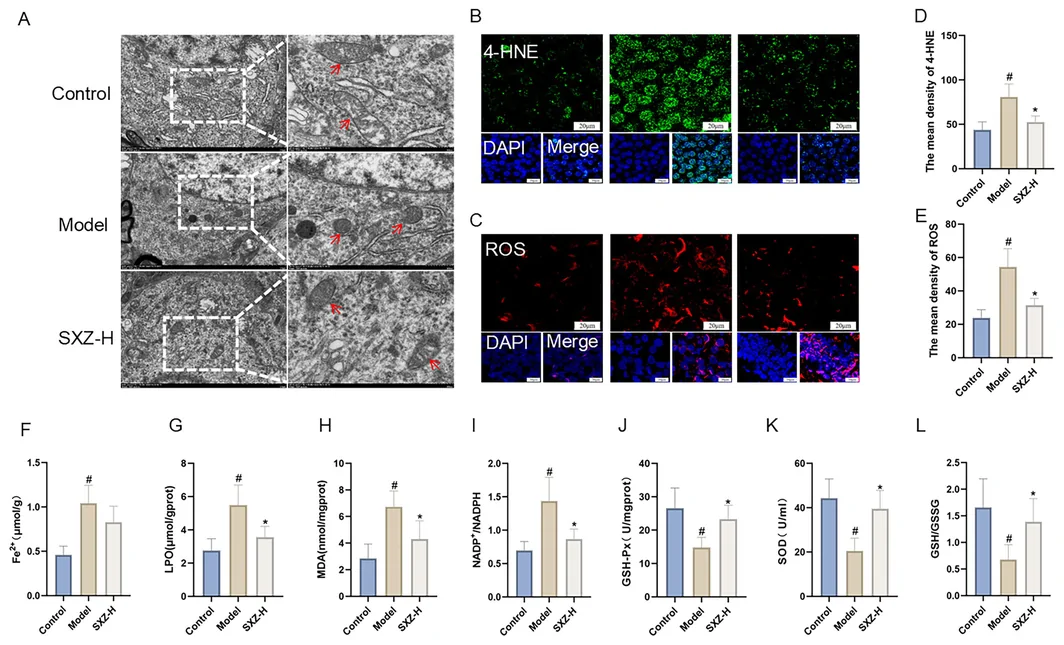
SXZ‐H inhibits ferroptosis in the hippocampus of naturally aging rats. (A) TEM images illustrating mitochondrial damage in hippocampal neurons. Red arrows indicate mitochondria; scale bars = 2 μm (left panel) and 500 nm (right panel). (B) Immunofluorescence images of 4‐HNE (100×, scale bar = 20 μm). (C) Quantification of 4‐HNE density (*n* = 3). (D) Immunofluorescence images of ROS (100×, scale bar = 20 μm). (E) Quantification of ROS density (*n* = 3). (F) Fe^2+^ levels (*n* = 6). (G) LPO levels (*n* = 6). (H) MDA levels (*n* = 6). (I) NADP^+^/NADPH ratio (*n* = 6). (J) GSH‐Px levels (*n* = 6). (K) SOD levels (*n* = 6). (L) GSH/GSSG ratio (*n* = 6). Data are presented as mean ± SD. ^#^ *p* < 0.05 versus Control; *p < 0.05 versus Model.

### 3.6 Effects of SXZ‐H on the Expression of Key Molecules in the p53/SLC7A11/GPX4 Pathway

Immunofluorescence staining revealed a significant increase in hippocampal p53 levels in the Model compared to the control group, whereas SLC7A11 and GPX4 expression levels were significantly diminished. The SXZ‐H treatment restored their expression after 6 months (Figure 5A,B). RT‐qPCR results and WB analysis consistently corroborated the same expression trends (Figure 5C–E). These results collectively imply that modulation of the p53/SLC7A11/GPX4 pathway may serve as a possible mechanism underlying the protective effect of SXZ‐H against age‐induced cognitive dysfunction.

**FIGURE 5:**
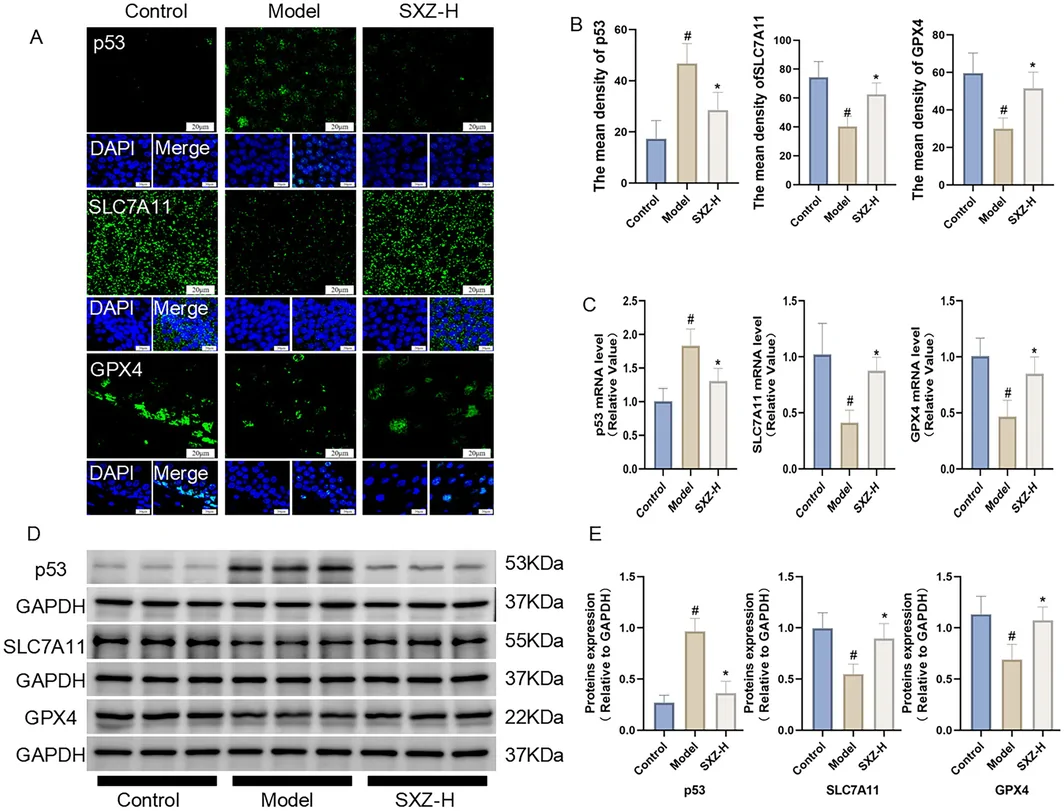
SXZ‐H modulates the p53/SLC7A11/GPX4 pathway. (A) Fluorescent staining of p53, SLC7A11, and GPX4 in the hippocampus (100×, scale bar = 20 μm). (B) Quantification of average density of p53, SLC7A11, and GPX4 (*n* = 3). (C) mRNA expression levels of p53, SLC7A11, and GPX4 in hippocampal tissue (*n* = 3). (D, E) WB analysis and quantification of p53, SLC7A11, and GPX4 protein expression (*n* = 3). Data are presented as mean ± SD. ^#^ *p* < 0.05 versus Control; *p < 0.05 versus Model.

### 3.7 Effects of SXZ‐H on the Expression of Key Molecules in the Keap1/Nrf2/HO‐1 Pathway

Immunofluorescence staining revealed a substantial increase in hippocampal Keap1 levels in the Model group compared to the control group, whereas Nrf2 and HO‐1 expression were significantly diminished. The SXZ‐H treatment restored their expression after 6 months (Figure 6A,B). RT‐qPCR and WB analysis consistently corroborated the same expression trends (Figure 6C–E). These results collectively suggest that modulation of the Keap1/Nrf2/HO‐1 pathway may underlie the protective effect of SXZ‐H against age‐induced cognitive dysfunction.

**FIGURE 6:**
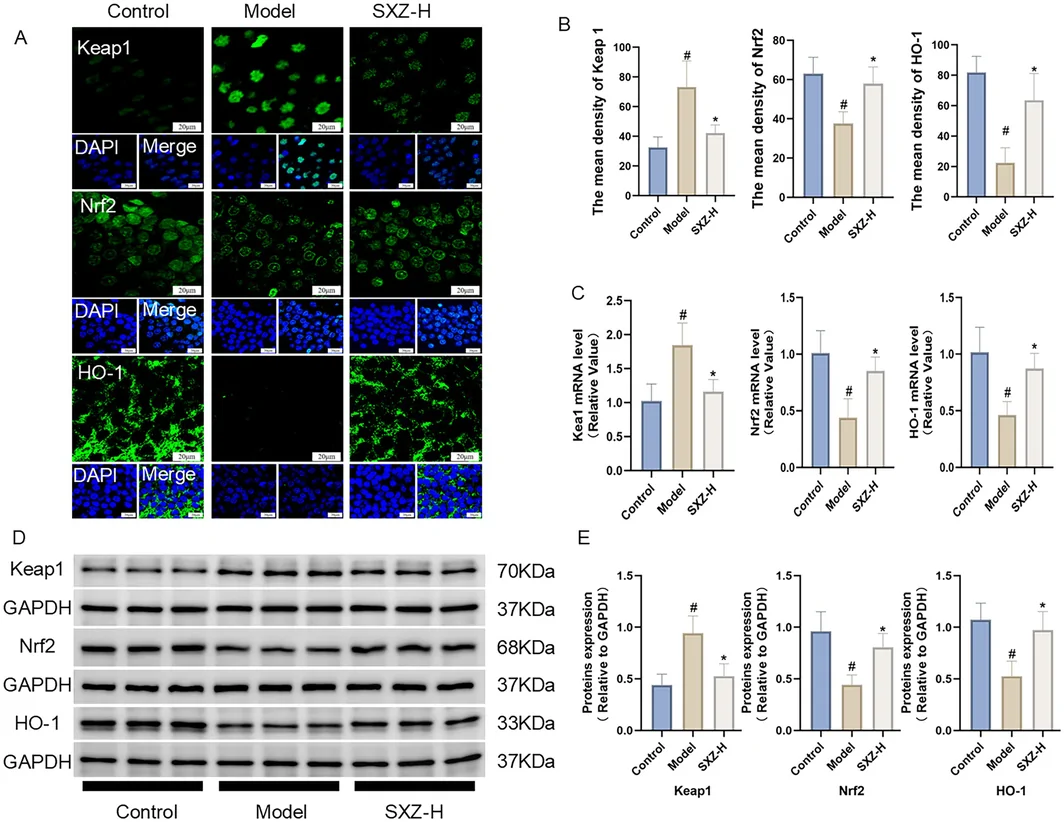
SXZ‐H modulates the Keap1/Nrf2/HO‐1 pathway. (A) Immunofluorescence staining of Keap1, Nrf2, and HO‐1 in the hippocampus (100×, scale bar = 20 μm). (B) Quantification of average density of Keap1, Nrf2, and HO‐1 (*n* = 3). (C) mRNA expression levels of Keap1, Nrf2, and HO‐1 in hippocampal tissue (*n* = 3). (D, E) WB analysis and quantification of Keap1, Nrf2, and HO‐1 protein expression (*n* = 3). Data are presented as mean ± SD. ^#^ *p* < 0.05 versus Control; *p < 0.05 versus Model.

## 4 Discussion and Conclusion

Aging represents a key contributor to neurodegenerative disorders and is a primary cause of decline in brain function [22]. Currently, D‐galactose is commonly used to induce aging in experimental animals, as it accelerates aging through nonenzymatic glycation reactions that generate advanced glycation end products. However, this model has limitations, as its pathological mechanism is relatively simple and lacks the chronic adaptive processes that naturally accumulate with age [23]. Additionally, sex differences significantly affect the progression of age‐related dementia, with women being more susceptible to severe cognitive impairments [24]. Previous studies have further demonstrated that microglial cells in women exhibit stronger pro‐inflammatory responses as they age [25]. Consequently, this study used 18‐month‐old female rats as a naturally aging model to more accurately mimic the aging process. Our analysis revealed clear differences between the Control group and Model groups, wherein 18‐month‐old rats exhibited significant impairments in cognitive function, as evidenced by prolonged escape latencies, diminished platform crossings, and modified duration in the target quadrant during the MWM test (Figure S2).

SXZ comprises *Oryza sativa* L., Dioscoreae Rhizoma, Allii Tuberosi Semen, and Euryales Semen. Previous studies have demonstrated that it possesses anti‐aging properties [12], and further evidence suggests that it can alleviate X‐ray‐induced cognitive impairment by modulating oxidative stress and inflammatory responses [13]. Here, we first identified the bioactive constituents of SXZ absorbed into the bloodstream using HPLC‐MS/MS. The results revealed that the major active compounds included lipids such as Pc(34:2); flavonoids, including Oroxylin A, Wogonin, Galangin 3‐methyl ether, Oroxyloside, and Sinensetin; and carbohydrates and their derivatives, including Paederoside and Verbasoside. Existing evidence has confirmed that Oroxylin A, a flavonoid compound, facilitates neurogenesis in the dentate gyrus of adult mice and enhances memory impairment [26]. Wogonin, another flavonoid, increases neuronal synaptic density in epileptic mice, ameliorates learning and memory deficits, and inhibits the release of pro‐inflammatory cytokines and microglia‐mediated synaptic phagocytosis [27]. Verbasoside, as a carbohydrate derivative, mitigates diabetic cognitive dysfunction in db/db mice by remodeling gut microbiota composition, enhancing metabolic disturbances, and reducing pathological brain damage [28]. Here, the Y‐maze and MWM were used to evaluate the learning and memory abilities under SXZ treatment in naturally aging rats, and three dose groups were set up: Low, medium, and high dose groups. Behavioral results revealed that all three dose groups dose‐dependently improved cognitive function in aging rats, with SXZ‐H having the most significant effect. SXZ‐H increased alternation behavior in the Y‐maze, reduced the latency to locate the hidden platform in the MWM, and enhanced the target quadrant dwell time and the number of platform crossings. These findings suggest that SXZ effectively ameliorated cognitive impairment in naturally aging rats, with SXZ‐H exhibiting the most pronounced impact. Notably, hippocampal neuronal function is closely associated with learning and memory capacity [29, 30]. Hence, we further examined the impact of SXZ‐H on the structural integrity of hippocampal neurons and used HE staining, Nissl staining, and NeuN immunohistochemistry for detection. The findings revealed that SXZ‐H improved hippocampal histopathology, increased Nissl^+^ neurons, and exerted a protective influence on hippocampal neurons in naturally aging rats. NeuN, a neuron‐specific nuclear protein, has been acknowledged as a reliable marker of mature neurons [31, 32]. Consistent with previous findings, a clear decline in NeuN expression in naturally aging rats was identified. However, SXZ‐H intervention significantly elevated NeuN expression in hippocampal neurons, indicating that SXZ‐H ameliorates pathological damage to hippocampal neurons in naturally aging rats. Based on the optimal results at high doses, the SXZ‐H group was selected for subsequent mechanism studies to further investigate its potential molecular pathways for improving cognitive function.

Metabolomics assessment revealed that natural aging significantly modified endogenous metabolites in the hippocampus of rats, and SXZ exerted a significant modulatory effect on these metabolic changes. Z‐score and KEGG enrichment analyses of differential metabolites further indicated that the protective effects of SXZ against age‐related cognitive impairment were primarily associated with glutathione metabolism and ferroptosis pathways. Visualization of the relative abundance of differential metabolites indicated that SXZ might exert its pharmacological effects by upregulating glutathionylspermine and downregulating Fe^2+^, glutathionylspermidine, iron, L‐ornithine, mevalonic acid, and NADP^+^. Previous studies have demonstrated that excess iron and Fe^2+^ can trigger neurodegeneration, and elevated iron concentration in AD reduces antioxidant defenses and alters mitochondrial function [33]. Similarly, during the development of cognitive impairment induced by chronic cerebral hypoperfusion, hippocampal NADPH/NADP^+^ levels are reduced in rats [34]. Glutathionylspermidine (Gsp), a derivative of glutathione (GSH), increases during elevated oxidative stress [35]. Additionally, coenzyme Q_10_ is associated with oxidative stress in the nervous system and cognitive impairment. Studies in older adults have confirmed that reduced CoQ_10_ levels are closely associated with the onset and progression of neurodegenerative diseases [36], findings that align with our untargeted metabolomics results. However, other studies have reported that L‐ornithine can alleviate hyperammonemia‐induced encephalopathy by reducing blood and brain ammonia concentrations [37]. Notably, in our untargeted metabolomics analysis, L‐ornithine levels were increased in the hippocampus of naturally aging rats, which may be attributed to disruptions in urea cycle‐related metabolic pathways, impairing its ammonia‐lowering and neuroprotective functions.

Ferroptosis is a regulated type of death marked by excessive lipid peroxidation and plays a pivotal role in neurodegenerative disorders, including cognitive impairment [38, 39]. Glutathione (GSH), a principal metabolite of glutathione metabolism, possesses potent antioxidant capacity and functions as a reducing agent to maintain the normal enzymatic activity of GPX4, which converts lipid peroxides (LPO) to their respective alcohols, thus mitigating ferroptosis [40]. In this study of physiological aging, hippocampal Fe^2+^, LPO, MDA, and 4‐HNE levels were significantly increased in aging rats, accompanied by marked lipid peroxidation and decreased GSH‐Px activity, which normally detoxifies LPO. Additionally, mitochondrial abnormalities marked by shrinkage, reduced size, increased membrane density, and decreased cristae were observed, indicating the onset of ferroptosis during aging. These findings align with those of Li J et al. [41], who reported elevated lipid peroxidation, mitochondrial shrinkage, and cristae degradation in hippocampal neurons of aging mice as hallmarks of ferroptosis. Additionally, we measured the GSH/GSSG ratio and observed a significant reduction in the hippocampus of aging rats, suggesting redox imbalance and further supporting the occurrence of ferroptosis. To corroborate this finding, we additionally assessed the NADP^+^/NADPH ratio, as NADPH functions as an electron donor to facilitate the glutathione reductase (GR)‐catalyzed reduction of GSSG to GSH [42]. Consistent with our findings, Ghosh D et al. [43], reported defects in neuronal NADPH levels, decreased NADPH‐generating capacity, and diminished GSH levels in aged non‐Tg and 3xTg‐AD mice. Notably, modern studies have identified iron, lipid, and amino acid metabolism as the three classic pathways of ferroptosis. This study found that the SXZ‐H intervention significantly reduced total iron and Fe^2+^ signals in the hippocampus of naturally aged rats. This implies that SXZ‐H could be involved in iron homeostasis regulation. However, in subsequent biochemical assays, the Fe^2+^ concentration in the hippocampus of naturally aged rats did not significantly decrease after 6 months of intervention. Studies indicate that the intracellular labile iron pool predominantly exists as glutathione‐iron(II) complexes (Fe^II^GS), rather than free Fe^2+^ [44]. Non‐targeted metabolomics might detect the “Fe^2+^” signal from bound iron molecules, such as glutathione, rather than from free ferrous ions. Iron in biological samples exists in multiple chemical forms, including ferritin‐bound iron, heme iron, and iron‐small molecule complexes, and precise identification of these iron species requires specialized detection methods [45, 46]. The reduction in the Fe^2+^ signal in metabolomics could suggest that SXZ‐H reduces iron affinity to specific metabolites, rather than directly lowering free iron concentrations. When combined with pathway enrichment analysis, we believe that SXZ‐H significantly modulates the glutathione metabolic pathway to enhance cellular antioxidant defense; conversely, it regulates lipid metabolic pathways associated with arachidonic acid metabolism and unsaturated fatty acid synthesis, thereby reducing the generation of lipid peroxides. The changes in the LPO assay contribute to this conclusion. Collectively, these findings suggest that natural aging induces lipid peroxidation in the rat hippocampus, leading to ferroptosis. However, SXZ‐H can effectively inhibit ferroptosis and improve cognitive function without directly altering the concentration of free Fe^2+^ through two pathways: Enhancing antioxidant defense and reducing lipid peroxidation substrates.

The tumor suppressor protein p53, recognized for its pivotal role in governing cellular senescence, also regulates ferroptosis through its impact on amino acid metabolic pathways. Specifically, p53 inhibits SLC7A11 transcription, thereby diminishing cystine uptake and GSH synthesis, leading to GPX4 inactivation, LPO accumulation, and ultimately triggering ferroptosis [47, 48]. Recent evidence suggests that p53 may serve as a potential biomarker for progressive neurodegenerative disorders, including AD, Parkinson's disease, and mild cognitive impairment [49]. Moreover, small ubiquitin‐like modifier (SUMO) modification of p53 accelerates AD‐related aging and tau pathology [50]. Chen W et al. [51], reported that neuronal death in the hippocampus of rats subjected to cerebral ischemia is associated with p53/SLC7A11‐mediated ferroptosis. Similarly, Ren X et al. [52], reported that p53 activation exacerbates cognitive deficits in AD mice and reduces SLC7A11 and GPX4 expression. Building upon previous reports, our study further confirmed that in the hippocampus of aging rats, p53 levels were significantly elevated, whereas SLC7A11, which imports cystine for GSH biosynthesis and antioxidant defense, and GPX4, which detoxifies LPO, were markedly reduced, as demonstrated by RT‐qPCR, WB, and immunofluorescence staining [53]. Notably, SXZ‐H treatment significantly downregulated p53 expression and restored SLC7A11 and GPX4 levels, indicating that SXZ may antagonize ferroptosis in the hippocampus of aging rats by suppressing p53 activation and enhancing SLC7A11 and GPX4 expression.

Ferroptosis is increasingly recognized as a process closely associated with oxidative stress, which acts as a fundamental mechanism underlying aging. Nuclear factor erythroid 2‐related factor 2 (Nrf2) is a central transcription factor that governs cellular antioxidant defense mechanisms. As oxidative stress levels rise, Nrf2 is released from its inhibitor Kelch‐like ECH‐associated protein 1 (Keap1), translocates to the nucleus, and subsequently activates the transcription of antioxidant genes, including HO‐1 and SOD [54, 55]. Beyond directly enhancing cellular antioxidant defenses, Nrf2 activation suppresses ferroptosis through multiple mechanisms, including the regulation of iron and lipid metabolism [56, 57]. Emerging evidence has implicated Nrf2 in aging and neurodegenerative diseases, including AD, Parkinson's disease, and stroke [58]. Yang S et al. [38], reported that Nrf2 activation attenuated cognitive deficits and inhibited neuronal ferroptosis in AD mice, and Yan N et al. [59], demonstrated that in rats with chronic cerebral hypoperfusion, impaired Nrf2 signaling resulted in decreased levels of downstream antioxidants, including SOD and HO‐1, accompanied by ferroptosis, which could be reversed by pharmacological intervention. Consistent with these findings, we observed increased ROS levels, reduced Nrf2 expression, increased expression of its negative regulator Keap1, and decreased HO‐1 levels in the hippocampus of naturally aging rats [60]. Furthermore, SOD activity, which is critical in scavenging ROS and counteracting oxidative stress, was diminished, along with dysregulation of ferroptosis‐related proteins. Notably, SXZ‐H treatment restored these alterations by promoting Nrf2 expression, mitigating oxidative stress in hippocampal neurons, and suppressing lipid peroxidation‐induced ferroptosis.

## 5 Limitations and Future Directions

This provides multi‐level, consistent evidence from behavioral assessment, histopathological examination, and untargeted metabolomic profiling. However, untargeted metabolomics, with some histological and immunofluorescence analyses, is inherently exploratory. Given the present experimental design, our hypothesis regarding glutathione metabolism and ferroptosis cannot be confirmed as a direct causal chain. Future research employing gene knockout or conditional overexpression techniques is essential to thoroughly confirm the necessity and sufficiency of these pathways in facilitating the cognitive‐enhancing effects of SXZ.

Furthermore, only three animals per group were incorporated for morphological assessment. This sample size is inadequate for robust statistical inference; thus, these findings should be viewed as preliminary and require further validation with larger cohorts in subsequent studies. Besides, as the experimental design was predominantly focused on cognitive‐behavioral assessment, we did not systematically collect serum biochemical indicators of liver and kidney function or perform corresponding histopathological examinations. Hence, we are unable to draw definitive conclusions regarding the systemic safety of long‐term high‐dose SXZ administration. During the experiment, daily observations confirmed a 100% survival rate across all groups, with no overt signs of toxicity. However, these observations can only indicate acute, severe toxic reactions and are insufficient for evaluating chronic systemic safety. Future studies should incorporate comprehensive biochemical and histopathological evaluations to address this limitation.

## 6 Conclusion

SXZ ameliorates cognitive impairment in naturally aged rats, as evidenced by improvements in behavioral performance and hippocampal histopathological morphology. Mechanistically, SXZ‐H regulates p53/SLC7A11/GPX4 and Keap1/Nrf2/GPX4 signaling pathways, thereby suppressing ferroptosis in the hippocampus. These findings suggest that SXZ‐H may counteract age‐related cognitive impairment by modulating pathways, potentially identifying new therapeutic targets and interventions for the clinical treatment of cognitive decline associated with aging.

## Author Contributions

Mengya Liu and Shunyi Li conceptualized and structured this paper. Mengya Liu, Yonglin Liang, and Yugui Zhang synthesized the literature and composed the article. Xutong Jia, Min Bai, and Yongqiang Duan amended the manuscript. All authors participated in the article's development and endorsed the submitted version.

## Funding

The financial support from Science and Technology Plan of Gansu Province [24JRRA565, 2023‐ZD‐236, zyzx‐2024‐zx15] facilitated the acquisition of essential resources, access to research facilities, and the execution of this study.

## Ethics Statement

All animal experiments conducted in this study were reviewed and approved by the Experimental Animal Ethics Committee of Gansu University of Traditional Chinese Medicine. Experimental Ethics No. SY2024–231, Animal Production License No. SCXK (Gan) 2021–0001, Animal Certificate No. 2437000310.

## Conflicts of Interest

The authors declare no conflicts of interest.

## Supporting information


**Data S1:** Supporting Information.

**Table S1:** Primer sequences.
**Table S2:** Names of identified bioactive compounds.

**Figure S1:** SXZ serum component profiles and classification. (A) Total ion chromatogram in positive mode; (B) Total ion chromatogram in negative mode; (C) Pie chart of constituent content distribution; (D) Pie chart of constituent number distribution.
**Figure S2:** MWM test. (A) Representative swimming paths recorded by the MWM system; (B) Latency to locate the platform (*n* = 12); (C) Duration in the target quadrant (*n* = 12); (D) Number of platform crossings (*n* = 12). Results are expressed as mean ± SEM. #p < 0.05 vs. Control group.

## Data Availability

The data that support the findings of this study are available from the corresponding author upon reasonable request.
