# Supplementary material for: Metabolomics Analysis Revealed That SXZ Ameliorates Cognitive Impairment in Naturally Aged Rats by Promoting Glutathione Metabolism and Inhibiting the Ferroptosis Pathway
Source: CNS Neurosci Ther. 2026 Oct 7;32(10):e71194. doi: 10.1002/cns.71194 (PMC13643645; doi:10.1002/cns.71194)
Supplement: Supplementary file 1 [file CNS-32-e71194-s001.docx]

1. SXZ Intervention Protocol

Shenxian Zhou (SXZ) was prepared from the following raw materials: Dioscoreae Rhizoma 40 g (Henan, 20210808), Euryales Semen 20 g (Guangdong, 20181206), Oryza sativa L. 30 g (Heilongjiang, C202310001), and Allii Tuberosi Semen 10 g (Gansu, 20191105). All herbs conformed to the specifications of Pharmacopoeia of the People’s Republic of China (2020 edition). Based on the preparation ratio documented in the postscript of the Dunhuang Manuscript P.3810 Hu Xi Jing Gong Miao Jue, herbs were weighed accordingly. The mixture was soaked in ten volumes of distilled water for 2 h and then decocted twice using standard procedures, each time for 40 min. After combining, the filtrates were concentrated by heating to obtain a preparation equivalent to 2 g of crude material in each mL. This decoction was preserved at 4 °C and adjusted to the necessary concentration before use.

2. Analysis of bioactive components of SXZ in serum

2.1 Preparation of SXZ medicated serum

Ten rats were randomly assigned to a control group and an SXZ treatment group, with five rats in each group. Rats in the SXZ group were administered 20 g/kg per day by gavage, divided into three daily doses, while rats in the control group received the same volume of pure water via gavage for three consecutive days. Two hours after the last administration, the rats were anesthetized and blood was collected via cardiac puncture. The blood samples were kept at room temperature for 1 h, then centrifuged at 3000 r/min for 15 min at 4 ℃ to obtain the serum supernatant^[1]^. Subsequently, the SXZ solution was filtered and sterilized using a 0.22 μm microporous membrane and stored in an ultra‑low temperature freezer at –80 ℃ for subsequent analysis of bioactive components.

2.2 HPLC-MS/MS Analysis

Each sample (100 μL) was added to 400 μL of extraction solution (1:1 acetonitrile:methanol) and vortexed for 30 s. Subsequently, after the samples were extracted, incubated, and separated, the supernatant was collected and transferred to vials for subsequent analysis. An UHPLC-Orbitrap Exploris 240 system (Thermo Fisher Scientific, USA) was used for the analysis. Chromatographic separation of the target polar metabolites was performed on an HSS T3 column (Waters, USA). The mobile phase consisted of solvent A (water/acetonitrile [95:5, v/v] with 0.1% formic acid) and solvent B (acetonitrile/isopropanol/water [47.5:47.5:5, v/v/v] with 0.1% formic acid), with a flow rate of 0.40 mL/min. Mass spectrometry was conducted with both primary and secondary data acquisition^[2, 3]^.

3. Behavioral Tests

2.3.1 Y-Maze Test for Spatial Memory

The Y-maze consisted of three identical opaque plastic arms, designated as the start arm, novel arm, and other arm. For the experiment, the arms were labeled as Arm 1, Arm 2, and Arm 3. The rats were introduced to Arm 1 and allowed unrestricted exploration for an 8-min period. An automated video tracking system recorded the total number of arm entries and the frequency of entries into each arm. The alternation index was calculated as: alternation number / (total arm entries - 2) × 100.

2.3.2 Evaluation of Learning and Memory Using the Morris Water Maze (MWM)

The MWM test was conducted by randomly placing the hidden platform at the center of one quadrant, with the platform submerged to a depth of 1 cm under the water surface. Titanium dioxide was added to the water to render it opaque, and the water was stirred evenly. Rats were placed in the southwest quadrant facing the pool wall, and the latency to reach the submerged platform was recorded. The rats were then dried and cared for. Each rat underwent four training trials per day, with the entry points located in different quadrants. Two trials were separated by 20 min, and the training procedure extended across five continuous days. On day 6, the platform was eliminated, and each rat was introduced into the quadrant farthest from the previous platform site. To assess spatial memory, the duration that rats remained in the target quadrant during the first minute was measured. Throughout the test, the video tracking system continuously monitored and collected behavioral data^[4]^.

4. Untargeted Metabolomics Testing and Analysis

2.1 Untargeted Metabolomics Testing

A Thermo Vanquish UHPLC instrument (Thermo Fisher Scientific, USA) was employed for metabolite separation. Chromatographic separation utilized an ACQUITY UPLC® HSS T3 column (1.8 μm, 2.1 × 100 mm; Waters, USA) set at 40 °C, with 2 μL injections at a flow rate of 0.3 mL/min. Mobile phases in positive ion mode were composed of 0.1% formic acid in water (A2) and in acetonitrile (B2). The elution gradient proceeded as: 0-1 min, 10% B2; 1-5 min, linear increase to 98% B2; 5-6.5 min, 98% B2; 6.5-6.6 min, decreased to 10% B2; and 6.6-8 min, held at 10% B2. In negative ion mode, water containing 5 mM ammonium formate (A3) and acetonitrile (B3) were employed under the same gradient conditions.

A Thermo Orbitrap Exploris 120 mass spectrometer (Thermo Fisher Scientific, USA) equipped with an ESI source was employed for detection in both positive and negative ion modes. The ion spray voltages were adjusted to +3.5 kV and -2.5 kV, while sheath and auxiliary gases were maintained at 40 and 10 arb, respectively. A capillary temperature of 325 °C was maintained. The mass spectrometer operated at 60,000 resolution for MS1 within an m/z window of 100-1000. Fragmentation employed HCD at 30% normalized collision energy, acquiring MS2 data at 15,000 resolution. Up to four precursor ions per cycle were fragmented, and dynamic exclusion was activated to prevent repeated selection.

2.2 Data Processing

Data from hippocampal tissue samples were processed using QI software. Raw data were first normalized by the total peak area normalization method, and missing values were imputed using the k‑nearest neighbor (KNN) algorithm. Metabolite features were annotated with a mass tolerance window of 15 ppm for precursor and fragment ions. Potential biomarkers were identified through unsupervised principal component analysis and supervised orthogonal partial least squares discriminant analysis (OPLS-DA). Selection criteria were set at *P* < 0.05 and VIP > 1. Quantitative metabolite data were normalized and visualized with the R package “Pheatmap,” and bidirectional clustering was performed for both samples and metabolites to generate hierarchical clustering heatmaps. Pathway enrichment analyses were conducted in combination with the Human Metabolome Database^[5]^, Kyoto Encyclopedia of Genes and Genomes (KEGG)^[6]^, and the in-house metabolite standards library of Nomics for comprehensive interpretation of metabolic alterations.

References

1.	Zhou CX, Cong W, Chen XQ, He SY, Elsheikha HM, Zhu XQ. Serum Metabolic Profiling of Oocyst-Induced Toxoplasma gondii Acute and Chronic Infections in Mice Using Mass-Spectrometry. Front Microbiol. 2017; 8: 2612.

2.	Jin Y, Cheng S, Liu R, Yu C, Zhang L, Li XL, Yan G, Zheng M, Zhe Min J. Comprehensive characterization of the chemical composition of Lurong dabu decoction and its absorbed prototypes and metabolites in rat plasma using UHPLC-Q Exactive Orbitrap-HRMS. Food Res Int. 2022; 161: 111852.

3.	Ren Y, Yu G, Shi C, Liu L, Guo Q, Han C, Zhang D, Zhang L, Liu B, Gao H, Zeng J, Zhou Y, Qiu Y, et al. Majorbio Cloud: A one-stop, comprehensive bioinformatic platform for multiomics analyses. Imeta. 2022; 1: e12.

4.	Chen Y, Q., Yang J, Pei WL, Han YM, Wang HP. Regulatory Effect of Hei Xiaoyaosan on Cognitive Impairment and Gut Microbiota in APP/PS1 Mice A. Chinese Journal of Experimental Traditional Medical Formulae. 2025; 31: 191-201.

5.	Wishart DS, Tzur D, Knox C, Eisner R, Guo AC, Young N, Cheng D, Jewell K, Arndt D, Sawhney S, Fung C, Nikolai L, Lewis M, et al. HMDB: the Human Metabolome Database. Nucleic Acids Res. 2007; 35: D521-6.

6.	Ogata H, Goto S, Sato K, Fujibuchi W, Bono H, Kanehisa M. KEGG: Kyoto Encyclopedia of Genes and Genomes. Nucleic Acids Res. 1999; 27: 29-34.
