# Supplementary material for: Metabolomics Analysis Revealed That SXZ Ameliorates Cognitive Impairment in Naturally Aged Rats by Promoting Glutathione Metabolism and Inhibiting the Ferroptosis Pathway
Source: CNS Neurosci Ther. 2026 Oct 7;32(10):e71194. doi: 10.1002/cns.71194 (PMC13643645; doi:10.1002/cns.71194)
Supplement: Supplementary file 2 [file CNS-32-e71194-s002.docx]

Figure S1. SXZ serum component profiles and classification. (A) Total ion chromatogram in positive mode; (B) Total ion chromatogram in negative mode; (C) Pie chart of constituent content distribution; (D) Pie chart of constituent number distribution.

Figure S2. MWM test. (A) Representative swimming paths recorded by the MWM system; (B) Latency to locate the platform (n = 12); (C) Duration in the target quadrant (n = 12); (D) Number of platform crossings (n = 12). Results are expressed as mean ± SEM. ^#^*P* < 0.05 vs. Control group.
