# Supplementary material for: Metabolomics Analysis Revealed That SXZ Ameliorates Cognitive Impairment in Naturally Aged Rats by Promoting Glutathione Metabolism and Inhibiting the Ferroptosis Pathway
Source: CNS Neurosci Ther. 2026 Oct 7;32(10):e71194. doi: 10.1002/cns.71194 (PMC13643645; doi:10.1002/cns.71194)
Supplement: Supplementary file 3 [file CNS-32-e71194-s003.docx]

**Table S1. Primer sequences**

| Name | Sequence（5'-3'） | Bp | Name | Sequence（5'-3'） | Bp |
| --- | --- | --- | --- | --- | --- |
| p53-F | 5'-TCCAGTTCATTGGGACTTATCCTTG-3' | 150 | Keap1-F | 5'-TGCTCGGGAGTATATCTACATGCAC-3' | 134 |
| p53-R | 5'-GCTCATATCCGACTGTGAATCCTC-3' | 150 | Keap1-R | 5'-CGATGCACGCATGGAACA-3' | 134 |
| SLC7A11-F | 5'-ATGTCCTGACAAATGTGGCCTA-3' | 131 | Nrf2-F | 5'-ATTGCCACCGCCAGGACTAC-3' | 78 |
| SLC7A11-R | 5'-GGCAACAAAGATCGGGACTG-3' | 131 | Nrf2-R | 5'-CCAAGATCTATGTCTTGCCTCCA-3' | 78 |
| GPX4-F | 5'-TTGCAGCCGGCTACAATGTC-3' | 174 | HO-1-F | 5'-GGCCTGCTAGCCTGGTTCAA-3' | 85 |
| GPX4-R | 5'-CCACGCAGCCGTTCTTATCA-3' | 174 | HO-1-R | 5'-GGTGTCTGGGATGAACTAGTGCTG-3' | 85 |
| GAPDH-F | 5'-GGCACAGTCAAGGCTGAGAATG-3' | 143 | GAPDH-R | 5'-ATGGTGGTGAAGACGCCAGTA-3' | 143 |

**Table S2. Names of identified bioactive compounds.**

| NO. | Metabolite | m/z | RT（min） | Mode | Adducts | Formula | Classification |
| --- | --- | --- | --- | --- | --- | --- | --- |
| 1 | Pc(34:2) | 758.568978 | 14.68758333 | pos | M+H | C_42_H_80_NO_8_P | A |
| 2 | Oroxylin A | 285.0755965 | 10.38225 | pos | M+H | C_16_H_12_O_5_ | B |
| 3 | Wogonin | 285.0756357 | 9.904466667 | pos | M+H | C_16_H_12_O_5_ | B |
| 4 | Galangin 3-methyl ether | 285.0755659 | 6.642583333 | pos | M+H | C_16_H_12_O_5_ | B |
| 5 | Methyl 3-(3,4-dihydroxyphenyl)propanoate | 201.0545536 | 5.439066667 | pos | M+Na-H_2_O | C_10_H_12_O_4_ | C |
| 6 | Paederoside | 447.0921082 | 5.90235 | pos | M+H | C_18_H_22_O_11_S | D |
| 7 | Oroxyloside | 461.1076849 | 6.642583333 | pos | M+H | C_22_H_20_O_11_ | B |
| 8 | Verbasoside | 461.1675 | 3.740283333 | neg | M-H | C_20_H_30_O_12_ | D |
| 9 | Sinensetin | 373.1280287 | 9.1396 | pos | M+H | C_20_H_20_O_7_ | B |
| 10 | 2-methyl-6-phenyl-4h-pyran-4-one | 187.075398 | 5.461033333 | pos | M+H | C_12_H_10_O_2_ | C |

Note: A: Lipids; B: Flavonoids; C: Others; D: Carbohydrates and derivatives.
